# Supplementary material for: LymphoTrack Is Equally Sensitive as PCR GeneScan and Sanger Sequencing for Detection of Clonal Rearrangements in ALL Patients
Source: Diagnostics (Basel). 2022 Jun 4;12(6):1389. doi: 10.3390/diagnostics12061389 (PMC9222020; doi:10.3390/diagnostics12061389)
Supplement: Supplementary file 1 [file diagnostics-12-01389-s001.zip › diagnostics-1730508-supplementary.pdf]

Supplementary Results

Correlation between RFU and %Total reads in samples with two or more rearrangements

IGH

In 26 of 28 samples (93%), the rearrangement that generated the highest RFU value correlated with the rearrangement that generated the highest %Total reads. A mismatch pattern was observed in sample 47 and 48. In sample 47, the peak with the highest RFU value demonstrated a VH1 rearrangement, whereas the VH1-JH1 rearrangement detected with LymphoTrack was assessed as the third most frequent rearrangement. In sample 48, the peak with the highest RFU value demonstrated a VH4-JH6 rearrangement, which was assessed as the second most frequent rearrangement with LymphoTrack (Supplementary Table 1). The discrepancies observed in two samples seemed to be caused by differences in amplification conditions in the different primer mixes.

IGK

In 15 of 19 samples (79%), the rearrangement that generated the highest RFU value correlated with the rearrangement that generated the highest %Total reads. A mismatch pattern was observed in sample 43, 50, 54 and 56, where the rearrangement with the highest RFU value was assessed as the second or third most frequent rearrangement in LymphoTrack (Supplementary Table 2). In the majority of the samples with 3 to 4 IGK rearrangements, the amplification efficiency differed between the IVS IGKA tube and the IGKB tube. E.g. in sample 55, the highest peak in the IGKB tube (14 200 RFU) generated only 6.2 %Total reads compared to the lowest peak in the IGKA tube (4 900 RFU) which generated 17.6 %Total reads. This mismatch pattern was also observed in samples 11, 43 and 56 (Supplementary Table 2). The discrepancies observed seemed to be caused by differences in amplification conditions in the IdentiClone tubes IGKA (complete rearrangements) and IGKB (incomplete rearrangements) compared to the LymphoTrack assay index master mix. In 4 of 6 samples, displaying 3 to 4 IGK rearrangements, a pattern was observed where primers amplifying

complete rearrangement seemed to outcompete primers for incomplete rearrangements with LymphoTrack. In order not to miss incomplete rearrangements suitable for MRD, we propose that confirmation using PCR GeneScan with the IVS IGKB tube should be used in cases where the LymphoTrack software identifies two complete rearrangements as the two most frequent rearrangements followed by one or two incomplete rearrangements. Alternatively, set a lower limit of %Total reads for clonality assessment in the interpretation criteria for the LymphoTrack IGK assay to include pertinent incomplete IGK rearrangements. However, to be able to determine such limitations, further comparisons on samples with 3 to 4 IGK rearrangements are needed.

TRG

In 26 of 41 samples (63%), the rearrangement that generated the highest RFU value correlated with the rearrangement that generated the highest %Total reads. A mismatch pattern was observed in sample 1, 3, 11, 20, 25, 27, 28, 37, 39, 41, 53, 59, 60, 74 and 76, where the rearrangement with the highest RFU value was assessed as the second or third most frequent rearrangement with LymphoTrack (Supplementary Table 3). Unfortunately, no clear pattern was observed, hence the discrepancies observed seemed to be caused by inexplicable differences regarding amplification conditions in the different BIOMED2 tubes compared to the index master mix in LymphoTrack. Analysis of amplification differences that might have been caused by variations in the primers targeting the Jγ-genes resulted in 8 of 12 (67%) samples where the BIOMED2 primer mix seemed to over-amplify rearrangements containing the Jγ1.3/2.3 gene. The LymphoTrack TRG assay detected eight rearrangements, which were absent in PCR GeneScan using the BIOMED2 GB tube. Seven of those rearrangements were subsequently confirmed with the IdentiClone® TCRG Gene Rearrangement Assay 2.0 for PCR GeneScan (IVS-1T-1F). This may indicate that the composition of primers and/or primer binding sites differs between the methods. The findings observed with the IVS-1T-1F may be explained by the inclusion of a new TRGJP primer. Despite this, it is not favorable using the IVS-1T-1F in PCR GeneScan prior to Sanger sequencing because of the single-color labeling

which makes it difficult to distinguish the different V- and Jγ-genes from each other (Armand, Derrieux et al. 2019).

TRB

In 7 of 13 samples (54%), the rearrangement that generated the highest RFU value correlated with the rearrangement that generated the highest %Total reads. A mismatch pattern was seen in sample 57, 61, 63, 69, 70 and 78, where the rearrangement with the highest RFU value was assessed as the second or third most frequent rearrangement with LymphoTrack (Supplementary Table 4). These discrepancies were expected to a certain degree since a comparatively large amount of primers, which were previously divided in three tubes (IVS) have been combined into one master mix in LymphoTrack, which may have led to other amplification conditions resulting in different amplification ratios.

Supplementary Table S1. Samples sequenced with LymphoTrack® Dx IGH FR1 Assay Panel - MiSeq®. The rearrangements, for each sample, are listed in falling order based on sequence frequency (%Total reads).

IGH								
Rearrangement GeneScan/Sanger				Rearrangement LymphoTrack				
**ID**	**V-gene**	**J-gene**	**RFU**	**Merge Count**	**V-gene**	**J-gene**	**%Total reads**	**Total Count**
**1**	VH3-66*	JH6*	7 100	758 455	IGHV3-66	IGHJ6	66.1	1 147 858
**2**	VH3-71*	JH4*	9 700	694 866	IGHV3-49	IGHJ4	80.9	858 649
**3**	VH6-1*	JH6*	30 000	240 827	IGHV6-1	IGHJ6	77.5	310 668
	VH6	NA	16 000	31 902	IGHV6-1	IGHJ6	10.3	
**4**	VH6-1*	JH6*	4 000	462 673	IGHV6-1	IGHJ6	77.3	598 489
**5**	VH3-73*	JH4*	10 600	1 051 922	IGHV3-73	IGHJ4	84.8	1 240 386
**6**	VH4-39*	JH6*	6 800	556 166	IGHV4-39	IGHJ6	84.7	656 898
	VH3-30*	JH4*	1 700	33 751	IGHV3-30	IGHJ4	5.1†	
**7**	VH2-26*	JH5*	2 400	89 621	IGHV2-26	IGHJ5	57.6	155 609
	VH1-69*	JH5*	1 400	6 742†	IGHV1-69	IGHJ5	4.3†	
**8**	VH3-11*	JH6*	32 500	2 558 156	IGHV3-11	IGHJ6	83.3	3 060 032
**9**	VH5-51*	JH6*	7 000	504 284	IGHV5-51	IGHJ6	37.5	1 344 772
**10**	VH3-30*	JH4*	13 400	99 350	IGHV3-30	IGHJ5‡	48.5	204 843
	VH4	NA	10 000	67 124	IGHV4-34	IGHJ6	32.8	
	VH4	NA	6 000	21 567	IGHV4-28	IGHJ5	10.5	
**11**	VH3-11*	JH4*	27 800	36 484	IGHV3-11_04	IGHJ4_02	53.6	68 054
	VH4-31*	JH5*	18 000	14 343	IGHV4-31_03	IGHJ5_02	21.1	
**12**	VH3-64*	JH4*	33 000	55 570	IGHV3-64	IGHJ4	89.6	62 021
**13**	VH3	NA	15 500	49 365	IGHV3-23	IGHJ6	94.7	52 149
**14**	VH3	NA	3 900	98 292	IGHV3-23	IGHJ4	15.4	640 286
**15**	VH3	NA	31 300	640 228	IGHV3-64	IGHJ4	76.1	841 107
**16**	VH5	NA	17 000	942 574	IGHV5-51	IGHJ6	83.3	1 131 091
**17**	VH3	NA	24 700	413 166	IGHV3-30	IGHJ6	46.1	895 378
	VH1	NA	21 000	225 518	IGHV1-3	IGHJ6	24.2	

**18**	VH6	NA	18 000	41 267	IGHV6-1	IGHJ4	75.4	54 747
**19**	VH3	NA	20 800	48 456	IGHV3-49	IGHJ4	89.4	54 196
**20**	VH3	NA	16 500	21 050	IGHV3-21	IGHJ6	39.4	53 471
	VH6	NA	11 000	13 617	IGHV6-1	IGHJ4	25.5	
**21**	VH4	NA	30 400	27 423	IGHV4-34	IGHJ6	53.1	51 664
	VH1	NA	18 700	16 925	IGHV1-2	IGHJ5	32.8	
	VH6	NA	3 500	1 258†	IGHV6-1	IGHJ6	2.4†	
**22**	VH6	NA	17 000	32 624	IGHV6-1	IGHJ6	54.7	59 652
	VH1	NA	11 000	19 220	IGHV1-3	IGHJ4	32.2	
**23**		POLYCLONAL			POLYCLONAL			
**24**		POLYCLONAL		733¥	IGHV3-7	IGHJ4	0.4¥	194 193
**25**	VH1	NA	16 600	398 217	IGHV1-2	IGHJ3	49.5	804 379
	VH2	NA	5 700	91 273	IGHV2-5	IGHJ3	11.3	
	VH1	NA	2 000	39 010	IGHV1-3	IGHJ3	4.8†	
	VH4	NA	1 900	32 861	IGHV4-34	IGHJ6	4.1†	
**26**	VH3	NA	18 000	511 321	IGHV3-74	IGHJ3	43.5	1 174 628
	VH3	NA	7 700	510 524	IGHV3-64	IGHJ4	43.5	
**27**	VH1	NA	32 300	1 092 897	IGHV1-3	IGHJ5	87.6	1 248 004
**28**	VH3	NA	30 000	729 389	IGHV3-11	IGHJ3	84.6	862 279
	VH1	NA	4 300	15 332	IGHV1-3	IGHJ5	1.8†	
**29**	VH4	NA	33 000	441 927	IGHV4-34	IGHJ6	43.4	1 019 290
	VH3	NA	29 500	429 762	IGHV3-35	IGHJ6	42.2	
**30**	VH4	NA	33 000	945 334	IGHV4-59	IGHJ6	86.6	1 092 251
	VH3	NA	1 300	2 294†	IGHV3-30	IGHJ6	0.2†	
**31**		POLYCLONAL		3 937¥	IGHV4-34	IGHJ4	2.3¥	172 182
**32**	VH3	NA	33 000	490 679	IGHV3-13	IGHJ4	45.8	1 072 240
	VH3	NA	28 000	421 918	IGHV3-30	IGHJ4	39.4	
**33**	VH3	NA	33 000	1 200 530	IGHV3-74	IGHJ5‡	69.1	1 738 748
	VH7	NA	22 000	332 161	IGHV7-4-1	IGHJ5‡	19.1	

**34**	VH3	NA	10 400	430 714	IGHV3-23	IGHJ6	29.3	1 472 567
**35**	VH1	NA	32 000	41 686	IGHV7-4-1	IGHJ4	53.9	77 438
	VH1	NA	24 000	26 511	IGHV1-69	IGHJ3	34.2	
**36**	VH1	NA	5 900	12 756	IGHV3-74	IGHJ6	26.9	47 365
	VH3	NA	3 400	7 271†	IGHV3-74	IGHJ6	15.4	
**37**	VH3	NA	30 000	28 712	IGHV3-30	IGHJ4	50.4	57 016
	VH3	NA	8 700	21 775	IGHV3-20	IGHJ6	38.2	
**38**	VH4	NA	29 600	399 652	IGHV4-34	IGHJ6	46.8	853 967
	VH2	NA	2 400	171 043	IGHV2-5	IGHJ4	20.0	
	VH3	NA	6 700	88 459	IGHV3-21	IGHJ6	10.4	
**39**		POLYCLONAL		348¥	IGHV6	IGHJ4	1.0¥	34 409
**40**	VH3	NA	12 800	60 618	IGHV3-13	IGHJ6	80.5	75 309
**41**	VH3	NA	11 300	50 006	IGHV3-30-3	IGHJ6	53.7	93 071
	VH2	NA	4 200	17 904	IGHV2-5	IGHJ5	19.2	
**42**	VH6	NA	5 400	72 725	IGHV6-1	IGHJ4	75.2	96 684
**43**	VH3	NA	14 400	57 628	IGHV3-9	IGHJ4	48.1	119 727
	VH3	NA	5 700	4 770†	IGHV3-11	IGHJ6	4.0†	
**44**	VH3	NA	26 200	92 192	IGHV3-43	IGHJ5	87.4	105 548
**45**	VH3	NA	7 800	58 464	IGHV3-30	IGHJ5	69.5	84 111
	VH2	NA	4 200	17 160	IGHV2-5	IGHJ6	20.4	
**46**	VH3	NA	17 000	15 451	IGHV3-d	IGHJ6	29.1	53 145
	VH3	NA	17 000	15 329	IGHV3-d	IGHJ6	28.8	
	VH3	NA	2 500	14 910	IGHV3-30	IGHJ5_02	28.1	
**47**	VH3	NA	25 100	314 704	IGHV3-66	IGHJ4	35.3	891 841
	VH3	NA	20 400	290 824	IGHV3-49	IGHJ1	32.6	
	VH1	NA	33 000£	136 095	IGHV1-69	IGHJ1	15.3	
**48**	VH3	NA	2 300	43 369	IGHV3-11	IGHJ2	56.5	76 776
	VH4	NA	5 500£	24 041	IGHV4-59	IGHJ6	31.3	
**49**	VH6	NA	7 600	34 100	IGHV6-1_02	IGHJ5_02	35.0	97 565
**50**		POLYCLONAL		46¥	IGHV3	IGHJ5	11.3	407¥

**51**	POLYCLONAL			1 295¥	IGHV6	none	4.5¥	28 944
**52**	VH3	NA	33 000	70 953	IGHV3-49	IGHJ6	91.8	77 307
**53**	VH6	NA	7 500	3 813 858	IGHV6-1	none	53.7	7 102 993
	VH6	NA	3 600	2 277 298	IGHV6-1	IGHJ4	32.1	
**54**	VH1-45*	JH6*	15 000	114 950	IGHV1-45	IGHJ6	53.9	213 135
**55**	VH3	NA	14 500	89 398	IGHV3-43	IGHJ6	71.7	124 685
	VH4	NA	11 800	24 063	IGHV4-34	IGHJ5	19.3	
**56**	VH3-23*	JH6*	19 000	427 753	IGHV3-23	IGHJ6	87.0	491 690

***Genes verified with Sanger Sequencing. The other genes were verified with GeneScan only, hence the NA in the J-gene column.**

NA Not Available.

†Interpretation criteria not fulfilled, and/or the peak observed with GeneScan is very small or has a high background.

‡The LymphoTrack Software states another gene family number than the IMGT database.

¥Interpretation criteria not fulfilled due to polyclonal pattern.

£The RFU value of the peak in GeneScan does not correlate to the amplification frequency %Total reads in LymphoTrack.

Supplementary Table S2. Samples sequenced with LymphoTrack® Dx IGK Assay Panel - MiSeq®. The rearrangements, for each sample, are listed in falling order based on sequence frequency (%Total reads).

IGK								
Rearrangement GeneScan/Sanger				Rearrangement LymphoTrack				
ID	V-gene	J-gene	RFU	Merge count	V-gene	J-gene	%Total reads	Total count
1	VK1-39*	JK1*	18 300	436 264	IGKV1D-39	IGKJ1	42.6	1 023 299
	VK2*	KDE*	4 000	191 167	IGKV2-30	IGKDEL	18.7	
2	VK6-21*	KDE*	6 000	489 962	IGKV6D-21	IGKDEL	60.1	815 044
	VK1f/6/VK7	KDE	1 000	159 881	IGKV7-3	IGKDEL	19.6	
3	VK1-33*	JK4*	21 000	422 140	IGKV1D-33	IGKJ4	45.1	935 954
	INTR*	KDE*	12 200	234 742	IGKINTR	IGKDEL	25.1	
4	VK3-15*	JK2*	33 000	564 363	IGKV3-15	IGKJ2	63.8	885 069
	VK3*	KDE*	10 400	230 884	IGKV3-7	IGKDEL	26.1	
5	VK1f/6/VK7	JK	32 500	260 417	IGKV1-16	IGKJ5	30.3	858 196
	INTR*	KDE*	13 300	238 602	IGKINTR	IGKDEL	27.8	
	VK2-30*	KDE*	7 300	135 158	IGKV2-30	IGKDEL	15.7	
6	POLYCLONAL			1 228¥	IGKV4-1	IGKJ4	4.2¥	29 060
7	VK3*	KDE*	23 200	329 589	IGKV3D-20	IGKDEL	39.4	837 174
	VK2*	KDE*	5 700	174 233	IGKV2-30	IGKDEL	20.8	
	INTR*	KDE*	400†	72 734	IGKINTR	IGKDEL	8.7†	
8	VK7-3*	KDE*	13 200	77 326	IGKV7-3	IGKDEL	9.8†	790 257
	VK1f/6/VK7	KDE	6 000†		ND			
9	VK1-5*	KDE*	32 500	485 012	IGKV1-5	IGKDEL	37.2	1 304 670
10	VK1f/6/VK7	JK	14 200	90 461	IGKV1D-37	IGKJ4	94.8	95 386
11	VK1f/6/VK7	JK	33 000	35 553	IGKV1D-37	IGKJ2	31.9	111 559
	VK2f/VK4/VK5	JK	5 200£	27 602	IGKV2D-26	IGKJ4	24.7	
	INTR*	KDE*	11 800£	20 398	IGKINTR	IGKDEL	18.3	
	VK2*	KDE*	3 600	11 196	IGKV2-24	IGKDEL	10.0	
12	VK1f/6/VK7*	KDE*	24 400	103 699	IGKV1-8	IGKDEL	33.6	308 672
13	VK1f/6/VK7	JK	23 000	138 845	IGKV1D-33	none	32.8	423 184
	POLYCLONAL			52 058	IGKINTR	IGKDEL	12.3	
	ND			41 802	IGKV1D-43	IGKJ2	10.0	

**14**	VK7*	KDE*	12 000	307 499	IGKV7-3	IGKDEL	25.5	1 205 279
	VK1f/6/VK7	KDE	6 800		ND			
**15**	VK2f/VK4/VK5	KDE	10 000	481 304	IGKV2-30	IGKDEL	47.3	1 016 655
**16**	VK3f	JK	20 000	1 448 397	IGKV3D-20	IGKJ2	76.9	1 883 420
**17**	VK1f/6/VK7	JK	16 500	923 153	IGKV1D-33	IGKJ4	83.5	1 105 869
**18**	POLYCLONAL			318¥	IGKV1-6	IGKJ2	7.1¥	4 507¥
**19**	POLYCLONAL			8 996¥	IGKV4-1	IGKJ2	9.6¥	93 695
**20**	VK2f/VK4/VK5	KDE	19 200	101 212	IGKV2-24	IGKDEL	70.3	143 935
**21**	POLYCLONAL			898¥	IGKV1	IGKJ2	3.0¥	30 145
**22**	POLYCLONAL			1 307¥	IGKV3D-20	IGKJ2	1.9¥	67 380
**23**	VK1f/6/VK7	KDE	1 300	34 104	IGKV1D-37	IGKDEL	8.0†	423 922
**24**	POLYCLONAL			2 783¥	IGKINTR	IGKDEL	0.6¥	474 912
**25**	VK3f	JK	19 500	357 833	IGKV3D-20	IGKJ4	44.3	807 487
	VK3f/INTR	KDE	6 000	164 354	IGKINTR	IGKDEL	20.4	
	VK3f/INTR	KDE	500	88 319	IGKINTR	IGKDEL	10.9	
**26**	VK1f/6/VK7	KDE	22 000	519 536	IGKV7-3	IGKDEL	45.0	1 154 919
	VK1f/6/VK7	KDE	7 200	477 179	IGKV1-16	IGKDEL	41.3	
**27**	VK1f/6/VK7	KDE	30 500	510 554	IGKV7-3	IGKDEL	41.6	1 228 807
	VK1f/6/VK7	KDE	7 100	505 842	IGKV7-3	IGKDEL	41.2	
**28**	VK3f	JK	33 000	1 136 199	IGKV3D-20	IGKJ2	69.2	1 642 863
	VK3f/INTR	KDE	33 000	421 355	IGKV3D-20	IGKDEL	25.7	
**29**	POLYCLONAL			6 054¥	IGKV1D-33	IGKJ4	1.4¥	432 512
**30**	VK3f	JK	12 500	778 373	IGKV3-15	IGKJ4	50.8	1 532 475
**31**	POLYCLONAL			31 581	IGKV5-2	none	5.4¥	581 270
**32**	POLYCLONAL			2 283¥	IGKV4-1	IGKJ2	2.8¥	82 223
**33**	VK3f/INTR	KDE	11 000	69 790	IGKV3D-20	IGKDEL	16.1	433 575

**34**	POLYCLONAL			16 852	IGKV7-3	IGKDEL	1.6¥	1 075 776
**35**	POLYCLONAL			2 592¥	IGKV3-15	IGKJ1	3.6¥	72 453
**36**	VK3f/INTR	KDE	23 000	6 981†	IGKINTR	IGKDEL	10.9	64 082
**37**	POLYCLONAL			452¥	IGKV4-1	IGKJ4	0.8¥	89 258
**38**	POLYCLONAL			1 890¥	IGKV3D-20	IGKDEL	2.6¥	72 067
**39**	VK2f/VK4/VK5	KDE	19 400	104 122	IGKV4-1	IGKDEL	81.4	127 847
**40**	POLYCLONAL			690¥	IGKV3	IGKJ1	3.5¥	19 875
**41**	POLYCLONAL			69¥	IGKV4-1	IGKDEL	4.8¥	1 425
**42**	POLYCLONAL			1 395¥	IGKV4-1	IGKJ4	1.9¥	73 449
**43**	VK2f/VK4/VK5	JK	3 300£	101 939	IGKV2D-30	none	43.3	235 579
	VK1f/6/VK7	JK	16 500	52 220	IGKV1D-39	IGKJ2	22.2	
	VK3f/INTR	KDE	19 700£	45 793	IGKINTR	IGKDEL	19.4	
**44**	VK1f/6/VK7	JK	27 800	91 117	IGKV1D-37	none	47.4	192 063
	VK3f/INTR	KDE	22 800	86 028	IGKV3D-20	IGKDEL	44.8	
**45**	POLYCLONAL			150 257	IGKV4-1	IGKJ4	2.7¥	5 448 611
**46**	POLYCLONAL			1 269¥	IGKV4	IGKJ2	1.3¥	96 174
**47**	VK1f/6/VK7	KDE	21 700	46 455	IGKV1-16	IGKDEL	76.5	60 730
**48**	VK2f/VK4/VK5	KDE	3 200	23 138	IGKV2-30	IGKDEL	53.9	42 902
**49**	POLYCLONAL			2 000¥	IGKV3D-20	IGKDEL	3.3¥	61 084
**50**	VK1f/6/VK7	JK	31 700£	22 822	IGKV1D-39	IGKJ2	47.5	48 057
	VK2f/VK4/VK5	KDE	33 000£	13 272	IGKV2D-30	IGKDEL	27.6	
**51**	VK1f/6/VK7	KDE	4 900	23 859	IGKV5	IGKJ2	4.2†	575 351
**52**	VK1f/6/VK7	KDE	25 600	32 899	IGKV7-3	IGKDEL	78.4	41 981
	VK1f/6/VK7	KDE	8 100		ND			
**53**	VK1f/6/VK7	JK	33 000	315 889	IGKV1D-39	IGKJ5	42.5	743 374
	VK3f/INTR	KDE	20 300	190 451	IGKINTR	IGKDEL	25.6	

**54**	POLYCLONAL			52 382	IGKV1D-33	IGKJ4	24.5	214 156
	VK3f/INTR	KDE	4 500	37 456	IGKINTR	IGKDEL	17.5	
	VK2f/VK4/VK5	KDE	2 000	22 332	IGKV2-30	IGKDEL	10.4	
**55**	VK3f	JK	33 000	118 222	IGKV3D-20	IGKJ2	69.0	171 471
	VK2f/VK4/VK5	JK	4 900£	30 104	IGKV2D-30	IGKJ4	17.6	
	INTR*	KDE*	14 200£	10 634	IGKINTR	IGKDEL	6.2†	
	VK2*	KDE*	7 100£	2 117†	IGKV2-30	IGKDEL	1.2†	
**56**	VK3f/INTR	KDE	9 400£	183 159	IGKINTR	IGKDEL	38.9	470 447
	VK2f/VK4/VK5	JK	33 000£	126 227	IGKV2-24	IGKJ5	26.8	
	VK2f/VK4/VK5	KDE	4 000	92 862	IGKV2-24	IGKDEL	19.7	

*Genes verified with Sanger Sequencing. The other genes were verified with GeneScan only.
ND Not Detected
†Interpretation criteria not fulfilled, and/or the peak observed with GeneScan is very small or has a high background.
¥Interpretation criteria not fulfilled due to polyclonal pattern.
£The RFU value of the peak in GeneScan does not correlate to the amplification frequency %Total reads in LymphoTrack.

Supplementary Table S3. Samples sequenced with LymphoTrack® Dx TRG Assay Panel - MiSeq®. The rearrangements, for each sample, are listed in falling order based on sequence frequency (%Total reads).

TCRG								
Rearrangement GeneScan/Sanger				Rearrangement LymphoTrack				
ID	V-gene	J-gene	RFU	Merge count	V-gene	J-gene	% total reads	Total count
1	Vγ2*	Jγ1.1/2.1*	28 000[£]	451 925	Vγ2	JγP1	53.2	850 268
	Vγ3*	Jγ1.3/2.3*	29 000[£]	269 801	Vγ3	none	31.7	
2	Vγ8*	Jγ1.1/2.1*	6 000	433 339	Vγ8	JγP1	43.4	997 995
	Vγ3*	Jγ1.3/2.3*	4 000	335 469	Vγ3	Jγ1/2	33.6	
3	Vγ9*	Jγ1.1/2.1*	13 600[£]	471 431	Vγ9	JγP1	56.6	833 048
	Vγ11[‡]	NA	17 000[£]	222 448	Vγ11	Jγ1/2	26.7	
4	Vγ11[‡]	NA	33 000	560 773	Vγ11	none	87.2	643 211
5	Vγ2*	Jγ1.1/2.1*	9 300	754 841	Vγ2	JγP1	67.4	1 119 271
6	Vγ9*	Jγ1.1/2.1*	1 100	41 176	Vγ9	none	15.9	259 130
	Vγ5*	Jγ1.3/2.3*	300	35 032	Vγ5	Jγ1/2	13.5	
7	Vγ2*	Jγ1.3/2.3*	2 200	431 140	Vγ2	Jγ1/2	57.1	754 597
	Vγ5*	Jγ1.3/2.3*	1 300	115 012	Vγ5	Jγ1/2	15.2	
8		POLYCLONAL		1 289	Vγ3	Jγ1/2	5.8¥	21 903
9	Vγ8	Jγ1.3/2.3	4 500	128 432	Vγ8	Jγ1/2	46.0	279 386
	Vγ5	Jγ1.3/2.3	4 500	110 325	Vγ5	none	39.5	
10	Vγ10	Jγ1.3/2.3	5 400	41 101	Vγ10	Jγ1/2	54.9	74 902
	Vγ2	Jγ1.3/2.3	4 000	30 128	Vγ2	Jγ1/2	40.2	
11	Vγ2*	Jγ1.3/2.3*	7 600	1 390 055	Vγ2	Jγ1/2	29.4	4 723 753
	Vγ1-8	Jγ1.1/2.1	1 800	1 936 503	Vγ3	JγP1	41.0	
12	Vγ1-8	Jγ1.1/2.1	4 100	8 124	Vγ3	JγP2	3.0¥	268 197
13		POLYCLONAL		5 664¥	Vγ9	Jγ1/2	9.7¥	58 171
14		POLYCLONAL		358¥	Vγ10	JγP1	0.6¥	60 501
15	Vγ1-8	Jγ1.1/2.1	14 000	1 586 018	Vγ8	JγP1	72.3	2 192 974
	Vγ9	Jγ1.3/2.3	10 000	327 210	Vγ9	Jγ1/2	14.9	

**16**		POLYCLONAL		6 072	Vγ2	Jγ1/2	3.4¥	180 326
**17**		POLYCLONAL		2 425	Vγ2	JγP2	1.6¥	153 365
**18**		POLYCLONAL		107¥	Vγ10	Jγ 1/2	4.8¥	2 248¥
**19**		POLYCLONAL		4 858¥	Vγ9	none	10.1	47 899
**20**	Vγ11‡	NA	15 000£	413 163	Vγ11	JγP1	53.6	771 393
	Vγ10	Jγ1.1/2.1	3 800£	210 673	Vγ10	JγP1	27.3	
	Vγ11‡	NA	11 000£	109 726	Vγ11	JγP1	14.2	
**21**		POLYCLONAL		387¥	Vγ11	Jγ1/2	1.8¥	21 502
**22**		POLYCLONAL		978¥	Vγ10	JγP1	2.2¥	45 156
**23**	Vγ9	Jγ1.1/2.1	33 000	219 519	Vγ9	JγP2	56.6	387 769
	Vγ9	Jγ1.3/2.3	1 800	100 656	Vγ9	Jγ1/2	26.0	
**24**		POLYCLONAL		2 810¥	Vγ11	JγP2	0.8¥	333 828
**25**	Vγ1-8	Jγ1.3/2.3	4 800£	208 793	Vγ3	Jγ1/2	57.5	363 208
	Vγ1-8	Jγ1.3/2.3	2 500£	101 484	Vγ2	Jγ1/2	27.9	
	Vγ9*	Jγ1.3/2.3*	8 300£	8 718	Vγ9	Jγ1/2	2.4†	
**26**	Vγ1-8	Jγ1.1/2.1	33 000	892 918	Vγ5	JγP1	69.4	1 287 512
	Vγ9	Jγ1.3/2.3	21 500	336 388	Vγ9	Jγ1/2	26.1	
**27**	Vγ1-8	Jγ1.1/2.1	8 400£	740 009	Vγ8	JγP1	55.5	1 332 525
	Vγ1-8	Jγ1.3/2.3	12 000£	497 602	Vγ2	Jγ1/2	37.3	
**28**	Vγ1-8	Jγ1.1/2.1	12 000£	361 680	Vγ8	JγP1	29.9	1 208 417
	Vγ1-8	Jγ1.3/2.3	22 300£	201 436	Vγ2	Jγ1/2	16.7	
**29**		POLYCLONAL		10 844	Vγ11	Jγ1/2	1.9¥	577 777
**30**		POLYCLONAL		1 114 568	Vγ2‡	JγP	89.4	1 246 423
**31**		POLYCLONAL		99 800	Vγ4	JγP1	8.9¥	1 116 539
**32**		POLYCLONAL		1 533¥	Vγ8	JγP1	2.0¥	76 887
**33**		POLYCLONAL		76 956	Vγ4	JγP1	6.9¥	1 116 832

**34**	Vγ1-8	Jγ1.3/2.3	5 200	374 907	Vγ4	none	35.4	1 057 985
**35**		POLYCLONAL		993¥	Vγ3	JγP2	1.7¥	59 490
**36**	Vγ1-8	Jγ1.1/2.1	9 800	377 749	Vγ8	JγP1	52.6	718 518
	Vγ1-8	Jγ1.1/2.1	3 100	112 863	Vγ8	JγP1	15.7	
**37**	Vγ1-8	Jγ1.1/2.1	6 500£	32 070	Vγ3	JγP1	60.3	53 216
	Vγ1-8	Jγ1.3/2.3	12 000£	19 351	Vγ3	Jγ1/2	36.4	
**38**	Vγ1-8	Jγ1.3/2.3	8 000	17 268	Vγ4	Jγ1/2	30.4	56 774
**39**	Vγ1-8	Jγ1.3/2.3	3 500£	262 224	Vγ4	Jγ1/2	49.0	534 950
	Vγ9	Jγ1.3/2.3	10 500£	71 499	Vγ9	Jγ1/2	13.4	
**40**		POLYCLONAL		184¥	Vγ2	JγP1	0.5¥	35 229
**41**	Vγ1-8	Jγ1.1/2.1	5 000£	28 348	Vγ3	none	58.1	48 769
	Vγ9	Jγ1.1/2.1	11 300£	17 570	Vγ9	JγP1	36.0	
**42**		POLYCLONAL		300¥	Vγ10	JγP1	1.6¥	19 345
**43**	Vγ9*	Jγ1.3/2.3*	2 800	9 180†	Vγ2	none	3.8†	239 131
**44**		POLYCLONAL		2 127¥	Vγ10	JγP1	1.4¥	155 491
**45**	Vγ11‡	NA	19 000	49 135	Vγ11	Jγ1/2	81.1	60 605
**46**	Vγ1-8	Jγ1.1/2.1	20 400	23 533	Vγ4	JγP1	52.2	45 060
	Vγ1-8	Jγ1.1/2.1	20 400	18 972	Vγ2	JγP2	42.1	
**47**	Vγ1-8	Jγ1.3/2.3	7 000	33 873	Vγ3	Jγ1/2	84.6	40 039
**48**	Vγ1-8	Jγ1.3/2.3	2 700	50 803	Vγ4	Jγ1/2	70.8	71 800
**49**	Vγ1-8*	Jγ1.3/2.3*	4 300	188†	Vγ5	Jγ1/2	11.6	1 620†
**50**	Vγ1-8	Jγ1.3/2.3	33 000	20 952	Vγ2	Jγ1/2	83.2	25 185
**51**		POLYCLONAL‡		26 047	Vγ11	JγP1	24.4	106 884
				15 787	Vγ11	JγP1	14.8	

**52**	Vγ1-8	Jγ1.3/2.3	10 500	559 282	Vγ4	none	63.9	874 948
	Vγ1-8	Jγ1.3/2.3	5 000	277 381	Vγ5	Jγ1/2	31.7	
**53**	Vγ1-8	Jγ1.1/2.1	500£	337 176	Vγ2	JγP1	56.9	592 517
	Vγ1-8	Jγ1.3/2.3	2 900£	230 765	Vγ4	Jγ1/2	39.0	
**54**	Vγ1-8	Jγ1.3/2.3	7 100	19 706	Vγ3	Jγ1/2	25.7	76 701
**55**	Vγ1-8	Jγ1.3/2.3	6 700	52 795	Vγ3	Jγ1/2	70.0	75 456
	Vγ1-8	Jγ1.3/2.3	5 600	19 831	Vγ2	Jγ1/2	26.3	
**56**	Vγ1-8	Jγ1.3/2.3	10 500	120 249	Vγ2	Jγ1/2	53.0	227 044
	Vγ9	Jγ1.3/2.3	10 500	47 124	Vγ9	Jγ1/2	20.8	
	Vγ9	Jγ1.3/2.3	4 100	25 222	Vγ9	Jγ1/2	11.1	
	Vγ1-8	Jγ1.3/2.3	600	21 179	Vγ3	Jγ1/2	9.3†	
**57**	Vγ2*	Jγ1.1/2.1*	33 000	689 925	Vγ2	JγP2	56.9	1 212 505
	Vγ2*	Jγ1.3/2.3*	19 000	403 883	Vγ2	Jγ1/2	33.3	
**58**	Vγ8*	Jγ1.1/2.1*	33 000	599 995	Vγ8	JγP2	61.8	970 476
	Vγ8	Jγ1.3/2.3	28 000	327 494	Vγ8	Jγ1/2	33.7	
**59**	Vγ8*	Jγ1.3/2.3*	3 400£	575 351	Vγ8	Jγ1/2	62.1	926 969
	Vγ9*	Jγ1.3/2.3*	6 000£	293 405	Vγ9	Jγ1/2	31.7	
**60**	Vγ5*	Jγ1.3/2.3*	9 000£	21 565	Vγ5	Jγ1/2	61.2	35 231
	Vγ9*	Jγ1.3/2.3*	10 000£	12 287	Vγ9	Jγ1/2	34.9	
**61**	Vγ10*	Jγ1.3/2.3*	7 000	453 471	Vγ10	Jγ1/2	49.1	924 069
	Vγ4*	Jγ1.3/2.3*	7 000	399 633	Vγ4	Jγ1/2	43.2	
**62**	Vγ4	Jγ1.3/2.3	5 000	686 996	Vγ4	Jγ1/2	59.3	1 159 138
	Vγ2	Jγ1.3/2.3	3 700	399 689	Vγ2	Jγ1/2	34.5	
**63**	Vγ4	Jγ1.3/2.3	27 000	182 699	Vγ4	Jγ1/2	74.4	245 581
	Vγ5	Jγ1.3/2.3	16 000	53 037	Vγ5	Jγ1/2	21.6	
**64**	Vγ4*	Jγ1.3/2.3*	13 300	3 155 792	Vγ4	Jγ1/2	51.0	6 184 779
	Vγ4	Jγ1.3/2.3	8 700	2 845 490	Vγ4	Jγ1/2	46.0	
**65**	Vγ4	Jγ1.3/2.3	33 000	50 434	Vγ4	Jγ1/2	50.5	99 944
	Vγ4	Jγ1.3/2.3	30 000	45 557	Vγ4	Jγ1/2	45.6	

**66**	Vγ2	Jγ1.1/2.1	33 000	39 315	Vγ2	JγP2	56.5	69 590
	Vγ4	Jγ1.3/2.3	4 500	26 880	Vγ4	Jγ1/2	38.6	
**67**	Vγ9*	Jγ1.1/2.1*	3 200	1 010†	Vγ2	Jγ1/2	3.0†	33 527
**68**		POLYCLONAL		386¥	Vγ10	JγP1	0.9¥	42945
**69**	Vγ1-8	Jγ1.3/2.3	3 500	148 375	Vγ3	Jγ1/2	43.5	341 468
	Vγ1-8	Jγ1.3/2.3	3 500	144 780	Vγ3	Jγ1/2	42.4	
**70**	Vγ10	Jγ1.3/2.3	5 000	197 588	Vγ10	Jγ1/2	51.2	385 827
	Vγ1-8	Jγ1.3/2.3	4 800	167 771	Vγ4	Jγ1/2	43.5	
**71**	Vγ1-8	Jγ1.3/2.3	9 300	26 231	Vγ2	Jγ1/2	49.9	52 606
	Vγ1-8	Jγ1.3/2.3	9 300	24 780	Vγ2	Jγ1/2	47.1	
**72**		POLYCLONAL		704¥	Vγ2	Jγ1/2	1.4¥	49 809
**73**	Vγ10	Jγ1.3/2.3	6 300	33 154	Vγ10	Jγ1/2	52.9	62 688
**74**	Vγ1-8	Jγ1.1/2.1	20 300£	363 418	Vγ8	JγP1	75.2	483 387
	Vγ9	Jγ1.3/2.3	24 600£	99 114	Vγ9	Jγ1/2	20.5	
**75**	Vγ9	Jγ1.3/2.3	13 500	179 078	Vγ9	Jγ1/2	41.4	432 308
	Vγ9	Jγ1.3/2.3	11 000	140 589	Vγ9	Jγ1/2	32.5	
**76**	Vγ1-8	Jγ1.1/2.1	1 800£	320 709	Vγ4	JγP1	70.9	452 639
	Vγ1-8	Jγ1.3/2.3	15 500£	116 733	Vγ2	Jγ1/2	25.8	
**77**	Vγ1-8	Jγ1.3/2.3	10 000	216 917	Vγ4	Jγ1/2	53.3	406 732
	Vγ1-8	Jγ1.3/2.3	6 300	144 117	Vγ3	Jγ1/2	35.4	
**78**	Vγ1-8	Jγ1.3/2.3	7 000	20 755	Vγ8	Jγ1/2	51.2	40 506
	Vγ1-8	Jγ1.3/2.3	6 000	18 657	Vγ5	Jγ1/2	46.1	

*Genes verified with Sanger Sequencing. The other genes were verified with GeneScan only, hence the NA in the J-gene column.
NA Not available
†Interpretation criteria not fulfilled, and/or the peak observed with GeneScan is very small or has a high background.
‡ The rearrangement(s) found with LymphoTrack were confirmed with qPCR and/or IVS TCRG 2.0 primer mix for PCR GeneScan.
¥Interpretation criteria not fulfilled due to polyclonal pattern.
£The RFU value of the peak in GeneScan does not correlate to the amplification frequency %Total reads in LymphoTrack.

Supplementary Table S4. Samples sequenced with LymphoTrack® Dx TRB Assay Panel - MiSeq®. The rearrangements, for each sample, are listed in falling order based on sequence frequency (%Total reads) from LymphoTrack.

TCRβ								
Rearranyement GeneScan/Sanger				Rearrangement LymphoTrack				
ID	V-gene/ D-gene	J-gene	RFU 1:0	Merge count	V-gene	J.gene	% total reads	Total count
**57**	Vβ5*	Jβ1.1*	1 000£	593 417	Vβ5-1	Jβ1-1	66.7	890 300
	Dβ1*	Jβ1.4*	8 800£	158 941	Dβ1	Jβ1-4	17.9	
**58**	VB6-6*	JB1.1*	8 700	597 583	Vβ6-6	none	47.5	1 258 658
	Vβ	JB1	4 500	320 188	Vβ6-6	none	25.4	
	VB6-2 or VB6-3*	JB2.5*	1 700	151 377	Vβ6-3‡	Jβ2-5	12.0	
**59**	Dβ1*	Jβ1.6*	33 000	945 638	Dβ1	Jβ1-6	60.2	1 572 017
	Vβ7-2*	Jβ2.7*	1 300	342 467	Vβ7-2	Jβ2-7	21.8	
**60**	Dβ2*	Jβ2.1*	15 000	36 669	Dβ2	Jβ2-1	42.3	86 637
	Dβ1*	Jβ1.6*	11 000	23 601	Dβ1	Jβ1-6	27.2	
	Dβ1	Jβ1	7 000	18 961	Dβ1	Jβ1-5	21.9	
**61**	Dβ1*	Jβ2.7*	3 300£	634 894	Dβ1	none	57.2	1 110 782
	Vβ20*	Jβ2.1*	11 800£	258 566	Vβ20-1	Jβ2-1	23.3	
	Vβ	Jβ1	3 000£	ND				
**62**	Vβ20-1*	Jβ2.1*	4 600	413 915	Vβ20-1	Jβ2-1	59.4	696 246
**63**	Vβ	Jβ1	16 000£	94 903	Vβ14	Jβ1-1	67.6	140 362
	Vβ10-1*	Jβ1-4*	2 500£	21 410	Vβ10-1	Jβ1-4	15.3	
	Dβ2*	Jβ2.1*	21 500£	18 043	Dβ2	Jβ2-1	12.9	
**64**	Vβ5-1*	Jβ1.5*	28 000	52 977	Vβ5-1	Jβ1-5	52.0	101 798
	Dβ1*	Jβ1.5*	18 000	41 155	Dβ1	Jβ1-5	40.4	
**65**	Data NA for TRB due to lack of DNA sample.							
**66**	Vβ12-5	Jβ2.1	24 600	60 300	Vβ12-5	Jβ2-1	51.0	118 307
	Dβ1*	Jβ1.4*	5 300	35 065	Dβ1	Jβ1-4	29.6	
**67**	POLYCLONAL			282¥	Vβ11-2	Jβ.3	2.4¥	11 611
**68**	POLYCLONAL			2 445¥	Dβ1	Jβ1.1	3.6¥	68 141

**69**	Vβ9	Jβ1.5	30 700£	174 866	Vβ9	Jβ1-5	39.8	439 808
	Dβ1*	Jβ2.1*	32 700£	135 827	Dβ1	Jβ2-1	30.9	
**70**	Dβ	Jβ1	7 300£	545 781	Dβ1	Jβ1-4	68.8	793 276
	Vβ	Jβ2	8 400£	74 730	Vβ7-7	Jβ2-1	9.4†	
**71**	Dβ2	Jβ2	33 000	75 230	Dβ2	Jβ2-3	54.2	138 756
	Vβ	Jβ2	33 000	50 861	Vβ27	Jβ2-2	36.7	
**72**		POLYCLONAL		53¥	Vβ18	Jβ1-2	0.8¥	6 989
**73**	Dβ2	Jβ2	4 000	17 387	Dβ2	Jβ2-1	9.2†	189 830
**74**	Dβ1	Jβ1	27 000	44 023	Dβ1	Jβ1-1	86.6	50 828
**75**	Dβ1	Jβ1	11 200	404 646	Dβ1	Jβ1-3	59.8	677 050
**76**	Vβ	Jβ1	29 300	441 918	Vβ10-1	Jβ1-4	63.5	695 786
	Dβ1	Jβ2	8 300	188 802	Dβ1	Jβ2-1	27.1	
**77**	Vβ	Jβ2	10 900	568 757	Vβ12-4	Jβ2-5	81.5	597 922
**78**	Dβ1	Jβ1	5 000£	37 303	Dβ1	Jβ1-1	33.4	111 579
		ND		35 905	Dβ1	Jβ1-2	32.2	
	Dβ2	Jβ2	22 000£	18 891	Dβ2	Jβ2-3	16.9	

*Genes verified with Sanger Sequencing. The other genes were verified with GeneScan only.
ND Not Detected
NA Not Available
†Interpretation criteria not fulfilled, and/or the peak observed with GeneScan is very small or has a high background.
‡The LymphoTrack Software states another gene family number than the IMGT database.
¥Interpretation criteria not fulfilled due to polyclonal pattern.
£The RFU value of the peak in GeneScan does not correlate to the amplification frequency %Total reads in LymphoTrack.
